# Does Policy Learning Meet the Standards of an Analytical Framework of the Policy Process?

**DOI:** 10.1111/psj.12250

**Published:** 2018-04-15

**Authors:** Claire A. Dunlop, Claudio M. Radaelli

**Keywords:** explanation, policy learning, causality, public policy, theories of the policy process

## Abstract

Reference to policy learning is commonplace in the public policy literature but the question of whether it qualifies as an analytical framework applicable to the policy process has yet to be systematically addressed. We therefore appraise learning as analytical framework in relation to four standards: assumptions and micro‐foundations, conceptual apparatus, observable implications, normative applications. We find that policy learning meets the four standards, although its theoretical leverage varies across them. Since we are not aware of theories of the policy process that meet all of these standards all the time, we conclude that policy learning fares reasonably well and it's worth investing intellectual resources in this field.

## 摘要

**政策学习能否作为政策过程的分析框架？:**

在公共政策文献中时常见到政策学习的参考资料, 但这样的参考资料是否能成为合格的分析框架, 应用于政策过程呢？这是还需要进行系统处理的问题。因此, 作者以四个标准评价了作为分析框架的政策学习。四个标准分别是：假设和微观基础、概念工具、可见的意义/影响, 以及规范性应用。作者发现, 政策学习达到了这四项标准, 尽管其在各项标准中的理论影响有所不同。由于本文并未发现能在任何时候都达到这些标准的政策过程理论, 结论认为, 政策学习是相当成功的, 并且值得对此领域投资智力资源。

## Resumen

**¿El aprendizaje político cumple con los estándares de un marco analítico en el proceso político?:**

La referencia al aprendizaje político es común en la literatura de la política pública, pero el interrogante de si esto califica como un marco analítico que se puede aplicar al proceso político todavía tiene que ser sistemáticamente abordado. Entonces evaluamos el aprendizaje como un marco analítico en relación con cuatro estándares: las suposiciones y las micro fundaciones; los aparatos conceptuales; las implicaciones observables; las aplicaciones normativas. Hallamos que el aprendizaje político cumple con los cuatro estándares, pero su ventaja teórica varía entre ellos. Ya que no estamos conscientes de teorías del proceso político que cumplan con todos estos estándares todo el tiempo, concluimos que el aprendizaje político se defiende razonablemente bien y tiene valor de inversión y recursos de inteligencia en este campo.

## Introduction and Motivation

Learning and its link to beliefs, policy development, and change is a central theme of public policy analysis. A recent review of the field found nearly one thousand political science articles dealing with topics of policy learning (Dunlop & Radaelli, 2013). In a recent version of the classic *Theories of the Policy Process* (Sabatier & Weible, 2014), learning as a causal mechanism is associated with most of the major policy frameworks outlined in the volume. We have found the causality of collective learning (Sabatier & Weible, 2014, p. 13, see also Heikkila & Gerlak, 2013), organizational learning within the multiple streams framework (Zahariadis, 2014, p. 44), policy‐oriented learning affecting social constructions in the context of the advocacy coalitions framework (Jenkins‐Smith, Nohrstedt, Weible, & Sabatier, 2014, p. 198), learning as a mechanism of policy diffusion (Berry & Berry, 2014, pp. 310–311), and learning as a meso‐theory adopted by the narrative policy framework (NPF) (Jones, Shanahan, & McBeth, 2014, p. 131). Since 2009, there have been five special issues devoted to learning in public policy journals—two on diffusion and transfer (Dolowitz, 2009; Evans, 2009, respectively); one on the EU as a learning organization (Zito & Schout, 2009); another on learning and policy failure (Dunlop, 2017a); a volume on policy change (Moyson, Scholten, & Weible, 2017) and another edited volume on modes and outcomes of policy learning (Dunlop, Radaelli & Trein, 2018)—all underpinned by international conference panels and workshops. In a nutshell, this is a growth field.

Given this interest, we raise the question of whether learning meets the standards of an analytical framework of the policy process. Our contribution to the literature is innovative because policy learning is either treated as a mechanism that supports other explanations or frameworks, or falls in the evaluation stage of the heuristic policy cycle (e.g., Araral, Fritzen, Howlett, Ramesh, & Wu, 2013). But in the literature, policy learning is not yet considered an analytical lens, as shown by its absence in all editions of *Theories of the Policy Process*, the advanced text pioneered by Paul Sabatier (Sabatier, 1999).

To answer our question, let us consider what a framework for the analysis of the policy process or lens “does.” Before we do that, let us bear in mind the distinction between theory and analytical framework (Carlsson, 2017; Dowding, 1995; George & Bennett, 2004, pp. 115–117; Stanley, 2012). Analytical frameworks contain simplifying ontological assumptions that are useful to understand the world and are applicable to a variety of research questions and contexts. Assumptions about learning should not be judged by their precision to reflect and match the world, but on how convincing and useful they are to categorize and reduce complexity—and to address certain research questions. The assumptions are stronger if they are derived from a theory, for example a theory of beliefs or a theory of the mind. Frameworks go beyond assumptions. They are used to generate and construct explanations or theoretical propositions (Stanley, 2012, p. 476), to normatively appraise a given phenomenon, and so on—but they are distinct from theories.

Theories explain and possibly predict a given reality, account for variance across their units and similarity in patterns, and finally allow us to generalize. We rehearse these points because the volume *Theories of the Policy Process* (Sabatier & Weible, 2014) actually contains “frameworks” as shown by the very label put on the advocacy coalitions framework (chapter 6), the narrative policy framework (chapter 7), the institutional analysis and development framework (chapter 8), and the multiple streams approach (chapter 2).

In policy analysis there is a shared understanding of what a framework should do, based on four criteria (Birchfield, 2013; Sabatier, 2007, p. 8; Zahariadis, 2013): (1) it should provide clear assumptions grounded in theory; (2) its concepts should have internal consistency and the main propositions should provide explanatory leverage; (3) it should contain observable implications—here is where the framework connects with theoretical propositions that can be tested; and (4) it should lead to normative appraisals of public policy, connecting the framework to democratic governance and reform.

We should be clear, none of the frameworks contained in *Theories of the Policy Process* is a total explainer. Indeed, we often hear that a multiple framework approach is better, whereby different perspectives can be layered to create wide explanations (Cairney & Heikkila, 2014). Granted that no framework can make claims to total and unique explanations, how does policy learning fare in relation to the four criteria?

A cursory overview of the field suggests a negative answer. Most articles routinely (and rightly) list the seminal work of Deutsch (1966), Lindblom (1965), and Heclo (1974). This is not simply giving due deference to the giants of our field, but also makes the point that very little has been built in terms of analytical framework in the intervening decades. Rather, policy learning is dominated by empirics sometimes organized around typologies (most obviously Bennett & Howlett, 1992, and May, 1992).

In the next section we review recent advances in the field, pointing to a set of concepts that is relevant for our discussion. The following sections examine the criteria to benchmark analytical frameworks, and the final section provides an answer to whether learning matches these criteria and where we go next. Throughout the article, we keep a focus on learning in the policy process, although we acknowledge that policy learning exists beyond the boundaries of the policy process.

## Looking for Conceptual Foundations to Build the Learning Framework

In recent years, political scientists began to look at learning again. Two sets of authors in particular have pushed the agenda—Heikkila and Gerlak (2013) on collective learning and Dunlop and Radaelli's (2013) varieties of learning. Here we focus on the model of varieties of learning. Heikkila and Gerlak (2013) provide a schema to distinguish between three elements: (1) where learning takes place—actor or system level; (2) the process and products of learning; and (3) within‐learning processes, between the acquisition of information, translation, and dissemination. This approach has a good deal of merit. It is a valuable way to break down learning into the three elements. In this contribution we do not have space to reflect on more than one approach, however. This time we opt for Dunlop and Radaelli's (2013) varieties of learning approach because it systematizes a large body of literature and suggests a congenial way to build an analytical framework. The authors carried out a bibliographic search, identifying an initial population of 833 articles on policy learning. After excluding duplicates, articles that refer to learning in purely descriptive ways and, entirely normative articles on “more” and “better” learning (see Dunlop & Radaelli, 2013, p. 616, footnote 2), their study explores in depth 83 articles that engage with learning as an analytical framework. On the basis of this sample, they identify two dimensions that map out the field into four main learning types. They then decompose the two‐by‐two space into sixteen subtypes. Contrary to other typological exercises, theirs draws explicitly on the method of explanatory typologies—a method that allows researchers to be clear about the causal architecture of their model (Elman, 2005). Thus, their typology is explanatory instead of descriptive—with learning types being the dependent variable that falls into the various cells of the types.

The full account of varieties of learning can be found in Dunlop and Radaelli (2013), but let us recall the essentials. Systematization of the policy learning literature in political science reveals four different learning processes, or modes, which recur empirically: epistemic, reflexive, bargaining, and hierarchical. These main learning modes are the product of two conditions associated with policymaking environments: the level of *tractability* and *certification of actors* associated with an issue. Next, to expand the property space to 16, they consider the variables of learners' control over the objectives of learning (high or low) and the learners' control over the content and means of learning (high or low). We outline this in the next section.

For the moment, let us stay within the two‐by‐two space of the higher‐level (of abstraction) typology, looking at tractability of the policy problem (Jenkins‐Smith, 1990) and social certification of actors (McAdam, Tarrow, & Tilly, 2001). Tractability, and its opposite (radical uncertainty), is prominent in the analysis of learning in systems of risk assessment, highly technical domains of environmental policy, policy instruments like regulatory impact assessment, and the social contestation of science. The point is simple: given high tractability, elected politicians and bureaucracies can define the pay‐offs associated with different courses of action. At the opposite, high or even radical uncertainty leads to reliance on epistemic communities, experts, and technical policy instruments. But, this variable is not limited to actors: it also refers to the forum or institutional setting of learning because highly tractable problems lend themselves quite naturally to standard operating procedures, technical fora, or delegation to independent regulatory agencies.

**Figure 1:**
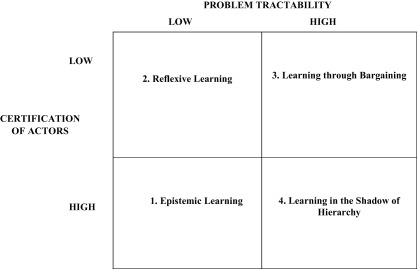
Conceptualizing Modes of Policy Learning.

The second dimension of variation across the literature is about who, in a given policy sector during a certain period, is socially certified as a teacher. This can be a central bank, an elected politician, or a nongovernmental organization like Amnesty International. Social certification can also direct toward specific institutional solutions, like a parliamentary committee or an inquiry. In short, certification concerns the extent to which a socially endorsed group or organization exists and has a seat at the policymaking table. In the absence of a privileged actor, learning participants will be plural—composed of a range of interested actors or of wider society itself. Taken together, levels of issue tractability and actor certification provide the axes for four types of policy learning to vary (see Figure 1).

This approach moves us beyond binary thinking. Its research questions are more fine‐grained than the presence or absence of learning. They are about the identification of a given type of learning, and whether over the course of time a policy sub‐system moves from one learning mode to another. The independent variables are dynamic—the level of issue tractability or actor certification adjusts to external developments and internal learning processes. Thus, the pre‐eminent mode of learning is in flux—over time, who creates the lessons that matter and the content of that learning will change. Finally, there are questions about whether an organization or a policy subsystem learns in the wrong mode, that is, dysfunctionally.

This way of mapping the literature is a promising start for tackling our research question about the status of policy learning. Establishing this status, however, requires a research design architecture able to account for causal relationships. And so, we move on to examine the criteria that an analytical framework should match.

## Clear Assumptions and Micro‐Foundations

To begin with, assumptions must be clear and grounded in theory. Without these, we have no axioms on which to build and are in danger of relying on common sense rather than scientific strategies (Sabatier, 2007, p. 5). Given the scale of the literature, we need a minimal definition of policy learning. Let us start from learning as an updating of beliefs about public policy. This matches the central concern of all policy analysis—the study of how beliefs inform policy debates; content; performance; institutional structures; and, on occasion, change. Beliefs are updated through social interaction, appraisals of one's experience or evidence‐based analysis, or most likely a mix of the three.

Yet, we are getting ahead of ourselves. What are the ontological assumptions that underpin this definition of learning and where do they come from? What are the micro‐foundations of policy learning? We need a micro‐level model of the individual that anchors our understanding of who learns, how, and with what effect. Here we enter the debate on micro‐foundations. This debate is played out in the territory of varieties of *homo economicus* or types of rationality—full or bounded. Recently, we have heard of *homo narrans* in the context of the narrative policy framework (NPF) (Jones & McBeth, 2010; Jones et al., 2014; Shanahan, Jones, McBeth, & Radaelli, 2018). Although the *homo economicus* can accommodate some types of learning, for example in game theory, this is a straightjacket: we need to identify a type of micro‐foundation sensitive to the vision of humans as sentient beings. Within learning as framework, *homo discentis*—the learning, studying, and practicing person—is at the heart of all policymaking. No matter what policy environment we operate in, what our role or standpoint, whether we work alone or in a collective, learning is the governing logic of action. Learning is how people make sense of the world.

The *homo discentis* vision of the individual is rooted in behavioral theories of psychology and adult education (Mocker & Spear, 1982; Rogers, 2002). We can think of this human as the composite of two sides. First, we have the *homo cognoscentis*. Our minds are full of prior knowledge, this knowledge is based on experiences, formal learning, intuitions, and values. These knowings are in a constant state of flux. We update and modify our beliefs as new information arrives. In line with the ground‐breaking work on belief systems in political science by Haas (1990), Hall (1993), Sabatier and Jenkins‐Smith (1993) and Muller (2000), these beliefs are of different types and have different degrees of resistance to change. For those beliefs most permeable to change, we do this on the basis of probability calculations—so‐called Bayesian reasoning—where we estimate the likelihood, or impact, of an event based on contextual conditions that we think are related to that event. Our second side is *homo doctrinis*—armed with these priors and updates we teach each other most frequently through argument or rhetoric; socialization; and, in some cases, coercion. Again, role and style of teaching are contextually contingent.

Yet, we do not assume that this updating is efficient or results in “correct” learning outcomes; far from it. When we update our knowledge and arguments—i.e., our learning—we do so on the basis of fragmented and incomplete information and with imperfect cognitive capacity (Jones, 2001). Uncertainty and complexity in the policy‐making environment and our own analytical limitations are the founding conditions of a world of “bounded rationality” (Simon, 1947, 1957). The goal here is to learn in ways that help us “satisfice” (Simon, 1956)—use what we know to find satisfactory solutions, given the limitations of the real world. In this view, the definition and understanding of policy problems is epistemically developmental and so temporally contingent. Thus, what we learn is inevitability fragile and filtered through “heuristics” or cognitive shortcuts that fill the void left by uncertainty (Jones, 2001; Kahneman, 2011). In his seminal text *The Art of Judgment* (1965), Vickers puts it thus: “[F]acts are relevant only in relation to some judgments of value and judgments of value are operative only in relation to some configuration of fact” (1965, p. 40). Policy action is a product of actors' judgments of their contextual reality and the cognitive biases they hold. Understanding policy learning, which may be functional or dysfunctional, is to recognize how these two realities intertwine to produce action and practices at any given moment.

These are still abstract notions for micro‐foundations—although not more abstract than the micro‐foundations of other frameworks—and remember what we said about assumptions in analytical frameworks: they should not be judged by how well they match reality. Anyhow, experimental studies provide supplementary knowledge on how individuals learn. Essentially we have two micro‐foundational mechanisms for individual learning. One is inferential, in the sense of drawing inferences by reasoning on what has happened. This reasoning has consequences for the priors and leads to an updating of beliefs. The drawing‐lessons operation can be cognitive or emotional, correct or incorrect. But inference is fundamental; hence this is inferential learning.

The other mechanism is called contingent learning (Kamkhaji & Radaelli, 2016). It occurs under conditions of extreme surprise and uncertainty. In these conditions, experimental studies have observed that individuals learn via fast‐paced associations of cue‐outcome dyads. Priors do not change, individuals do not choose behavior on the basis of their understanding of cause‐effect relations. They do not reason inferentially on what has happened. Surprise throws in a set of unexpected cue–outcome relations—decision makers are typically confronting these relations in crisis‐related episodes. It is exactly the lack of experience about the relationship existing between a given stimulus and an outcome that triggers the mechanism. This surprise about the causal relationship generates contingent learning. Once contingent learning is triggered, feedback and the passing of time create the basis for inferential learning, where individuals understand, decode, and learn inferentially what they have done before in contingent fashion. Thus, under conditions of crisis and extreme surprise, we have micro‐foundations that illustrate the sequence between contingent learning and inferential learning. This sequence has been successfully probed in the context of the crisis of the Euro—showing that the findings under carefully controlled experiments can also apply to the domain of public policy (Kamkhaji & Radaelli, 2016).

## Logically Consistent Concepts and Clear Causal Drivers

Beyond clarity around its starting assumptions, an analytical framework must be internally consistent and offer clear causal drivers (Sabatier, 1999, p. 8). We observe two elements that ensure coherence. One is the fact that the concept of learning must cover the full spectrum, from unlearning to zero learning to learning (Radaelli, 2009). This is a property that is fundamental to avoid bias in empirical analysis, where the risk is one of censoring learning as a variable by looking only at its positive values. The other is to allow learning to vary empirically from enlightenment to endarkenment (Weiss, 1977, 1979, and more recently Daviter, 2015). We shall go back to this second element later on—we flag it now because it is fundamental for conceptual consistency.

To explore in depth the conceptual and causal architecture of policy learning, we need to go back to the four varieties of learning. The varieties are distinguished by: knowledge use, the causal mechanisms that underpin that use, actors' modes of interaction, decision makers' mode of attention, the benefits they bring when learning is functional, and the “pathologies” or degenerations that may result from poor learning performances (see Table 1).

**Table 1:** Unpacking Varieties of Learning

Learning as …	Epistemic	Reflexive	Bargaining	Hierarchical
Knowledge use as …	instrumental	conceptual	political/symbolic	imposed
Causal mechanism …	expert teaching	deliberation	resource competition	institutional rules
Interaction of policy actors as …	cooperative asymmetric	cooperative symmetric	competitive symmetric	competitive asymmetric
Benefits as …	clinching what works	depth of debate and breadth of knowledge types	wide range of evidence scanned	locks‐in evidence
Pathologies as …	groupthink	uneven capacity leads to spurious consensus	unstable outcomes and expert discrediting or withdrawal	blocked learning and expert defeatism
Decision makers' attention as …	directed	diffuse/divided	selective	routinized
Mode underpinned by a logic of …	rationality	appropriateness	consequence	habit

Source: Dunlop, 2014 (Tables 1 and 2, pp. 212, 216).

1. *Epistemic learning* takes place where knowledge is created around an issue with low tractability by a certified set of experts. The archetypal actor is the expert, or, collectively, the epistemic community with participatory or consultative rights in policy processes where complexity has to be negotiated and ultimately reduced. Epistemic actors occasionally enjoy direct policymaking responsibilities, such as the Delors Committee that paved the way for the decision to create the Euro (Verdun, 1999) or the European Central Bank in circumstances of radical uncertainty during the Euro crisis of 2010–2011. To use Weiss's (1979) famous typology, knowledge use is instrumental—the aim is for experts' advice to show up directly in policy development. Such impact is achieved by the close relationship that can develop between decision makers and epistemic communities. Knowledge deficits on the part of decision makers ensures this mode of interaction is necessarily asymmetrical, with decision makers effectively being taught by the experts. For example, under conditions of radical uncertainty or where a group of experts is particularly revered, decision makers' attention is “directed” to what they say and can result in expert involvement in preference formation (see Haas, 1990).
2. *Reflexive learning* occurs where uncertainty is at its highest—the issue lacks tractability and there is no socially agreed set of actors who is certified as the teacher. Instead, we have deliberation and contestation by a pluralist set of actors. Accordingly, knowledge use is conceptual—there to fuel and then consolidate the results of a socially thick policy conversation. In such uncertain policy environments, outcomes are difficult to predict but exchanges are cooperative. In its ideal form, this reflexive learning has Habermasian qualities—where decision makers' attention is diffused as they engage with a wide range of actors and viewpoints. Policy learning occurs over time through communication, preference change, and collective puzzling. Here, the technocratic world of epistemic learning dominated by codified knowledge is replaced with a wide range of knowledge types. Tacit, uncodified knowledge—such as myths or innuendo—take their place alongside formal knowledge in informing and changing beliefs about the appropriate form of policy (Sanderson, 2002). In its functional manifestations, we have the wisdom of crowds. Yet, the flip side of this is the so‐called myth of the best argument (Pellizoni, 2001), where incommensurable paradigms should be acknowledged or else risk a fake consensus in the name of the “best argument.”
3. *Learning through bargaining* captures situations where learning occurs as an unintended, but nonetheless potent, by‐product of interest‐driven stakeholders. Where actor certification and uncertainty are both low, learning takes place through competitive interactions of stakeholders who select evidence from a range of “knowers” that suits their policy preferences. While knowledge use is political or symbolic, policy‐based evidence need not be a negative phenomenon. The polyarchic nature of these interactions ensures that a wide range of evidence may be aired in policy debates. Moreover, the stability generated by processes of partisan mutual adjustment (Lindblom, 1965) may ensure that some lessons hold for long periods. Yet, where evidence is judged to have been manipulated to secure policy preferences, learning through bargaining may degenerate resulting in a retreat of and from ideas.
4. *Learning in the shadow of hierarchy* occurs in hierarchical contexts where authorities use the vertical organization of policymaking to force knowledge use. Such imposition (captured in Weiss's later work—Weiss, Murphy‐Brown, & Birkeland, 2005) is a result of circumstances where learning is structured by vertical institutional rules, for example rules that discipline budget constraints within which social policy can be made, or rules about intergovernmental relations. Interactions here are governed by a habitual logic (Hopf, 2010). Institutions use their mandates to steer learning from the “top” (Radaelli, 2008) in ways that minimize negotiations and exceptions to the rules. Learning becomes an exercise in gathering information centrally, and translating and disseminating it via instructions supported by incentives and sanctions. For the recipients at the bottom, learning is about understanding what is expected from them, the nature of instructions, and the forms of compliance. Thus, there is learning about compliance on a substantive topic, and meta‐learning about understanding the ecology of rules and how the authorities expect the recipients to use them.

**Figure 2:**
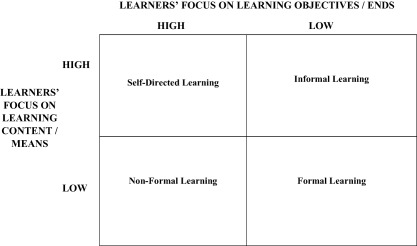
Mocker and Spear's Lifelong Learning Typology.

**Figure 3:**
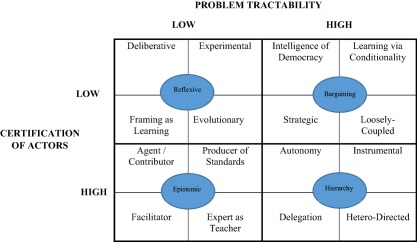
Expanding Modes of Policy Learning.

To investigate the causal architecture further, Dunlop and Radaelli (2013, pp. 604–613) expand the property space of each of our four learning types with a theory of adult learning that differentiates between situations where learners focus on the substance or goal of knowledge creation and communication. High or low focus over what we learn and why results in four adult learning types: self‐directed learning, informal learning, non‐formal learning, and formal learning (Mocker & Spear, 1982; see Figure 2). These types are constructed on empirical reality and assumptions of intended rationality. In line with the framework construction outlined already, this yields an account of learning dynamics which are objectively probable and involve a low degree of abstraction. We end up with four roles for *each cell* of the basic model of Figure 1. Thus, in total the literature can be mapped out in 16 learning modes from the original four (Figure 3).

At any one time, we expect that a learning mode will dominate policymaking around an issue. As noted earlier, the mode that matters is contingent upon the state of our two dimensions. Changes in issue tractability and/or actor certification will trigger moves within and between learning types. The conditions are not simply set objective realities that exist “out there.” Rather, they are constructed by decision makers in government who steer governance on the basis of exogenous conditions—economic performance, legal protocols, political power shifts—or endogenous developments—most notably learning and unlearning by policy actors.

In terms of applicability, there is no one‐to‐one necessary correspondence between learning and a given stage of the policy process. While it is likely that epistemic learning may be confined to the issue framing stages—where uncertainty is at its peak—the model does not set this restriction. There is no *conceptual* reason that the epistemic mode cannot characterize implementation, though empirically this may be less likely than agenda‐setting. In common with most policy frameworks, we assume liberal democratic politics with a civil society infrastructure. With that in place, the framework does not favor any particular political system over another.

To wrap up, we have demonstrated learning as an analytical framework is anchored to a set of coherent concepts and a causal architecture based on explanatory typologies. The learning approach we have described mitigates bias by allowing all values of the variables, without censoring some. Further, the explanatory typology is grounded in robust theories: adult education and policy theory. The 16‐role expansion shows the connections between different types, and identifies the drivers that take us from one cell in Figure 3 to the others. To see whether all this amounts to explanatory leverage, we now move to the next section.

## Implications and Empirical Testing

Learning can assist researchers in the field in three ways that relate to observable implications, but with different features.

The first pathway is observable implications that contribute to existing frameworks on the policy process. To illustrate: the varieties of learning framework can feed into the development of the narrative policy framework by putting forward propositions like “if learning is of type X, then narratives and narrators will have these observable characteristics Y_1_ and Y_2_.” By way of exemplification, in Table 2, we portray the observable implications of learning types for a few of the key variables that define the focus of the narrative policy framework (NPF). This is just a suggested formulation of observable implications for the NPF. Others can be added—for example on the features of the causal plot, with epistemic‐led narrative plot anchored to the authority of science and evidence whilst a hierarchical causal plot will emphasize law, formal authority, and due procedure.

**Table 2:** Varieties of Learning: Observable Implications for the Narrative Policy Framework

NPF Categories	Epistemic	Reflexive	Bargaining	Hierarchical
Narrator is defined on the basis of her …	knowledge	argument and ethics	resources	formal authority
Decision maker is …	teacher	maieutic agent	facilitator of exchanges	interpreter and developer of formal rules
Policy problems are narratively represented as …	information and know‐how problems	mutual obligations	benefit‐cost issues	procedures and targets
Drama and metaphors	emphasizing the value of research and knowledge	pushing toward mutual understanding and collective binding	magnifying advantages from social exchange	illustrating consequences of compliance and lack thereof
Heroes and villains	heroes proceed via enlightenment	heroes respect social norms	villains are those who do not sit at the table and do not accept to negotiate	embedded in conceptions of compliance with rules
Doomsday scenarios explain the consequences of …	acting in irrational ways	rejecting a community based on deliberation and common fate	following short‐term interests	lack of compliance, sanctions, and punishment

We can do the same for the advocacy coalition framework (ACF)—one of the most established and tested frameworks. The ACF assists the development of observable implications for the learning framework as much as the latter enriches the ACF. The ACF central questions relate to the varieties of learning. When does policy learning (or policy‐oriented learning to use ACF language) lead to policy change? What is it that changes? Three features of the policy subsystem matter in explaining the context and events that foster learning: the nature of professional fora, level of conflict, and the analytical tractability of the issue (Jenkins‐Smith, 1990, pp. 95–103; Sabatier, 1987, pp. 678–681; Weible & Nohrstedt, 2013, pp. 130–131). These characteristics affect the object of learning—i.e., the likelihood that policy core or secondary beliefs will be altered. We now relate these conditions to the varieties of learning.

In his discussion of professional fora, Jenkins‐Smith (1990, pp. 99–103) emphasizes the imprint left by the level of conflict, analytical tractability, and type of forum on the use of analysis. A focus on learning types extends this type of reasoning to capture the wider effects of social science utilization. Let us see how, starting from the type of forum. Since the forum disciplines participation, we can have an open forum, a professionalized forum, or a closed forum. Professionalized settings are characterized by shared analytical training and norms. Consequently, access to debate is screened. The likelihood of using social sciences to learn is high. A professionalized forum matches epistemic learning environments. Hierarchical settings discipline access by drawing on formal rules (of representation, for example) and levels of governance. Professionalized fora can facilitate learning by providing know how in relation to clearly specified goals and targets. Learning is more exploratory in reflexivity mode, where professionalized fora do not necessarily have a pivotal position. Quite the contrary, they may be marginalized because professional and lay knowledge have the same value. In bargaining, professionalized fora are captured by partisan mutual adjustment—they can be instrumental and effective if they accept to become a component of the advocacy game.

The second condition concerns the level of conflict between actors in learning modes (Jenkins‐Smith, 1990, pp. 95–97; Weible, 2008). Learning is frustrated where conflict is very low—hierarchical settings—and the incentives to update policy core and deep beliefs are absent. Conversely, in reflexive settings conflict is transparent but can be extreme. Reflexivity makes the assumption that communicative rationality assists one coalition in overcoming defensive positions and in accepting to hear the argument of the other coalition. Whether policy processes approximate these ideal‐speech situations is another story. This is why in ACF analysis, the learning “sweet spot” is found where there are intermediate levels of conflict—settings where “there is enough of a threat to attract the attention of rivals but not too much of a threat to entrench opponents on rigid policy positions” (Weible & Nohrstedt, 2013, p. 131). Learning through bargaining and in epistemic mode best fit this description.

Finally, ACF analysis treats learning as conditioned by the level of analytical tractability of an issue (Jenkins‐Smith, 1990, pp. 97–99)—we have already discussed this variable when we presented Dunlop and Radaelli (2013).

Varieties of learning assists the ACF in qualifying the types of learning, and within each type, the causal mechanisms that drive learning. It also allows for more endogenous forms of learning within and across coalitions (especially in the reflexive and bargaining modes), whereas the ACF is more powerful in explaining learning triggered by variables that are exogenous to the policy‐subsystem.

The second pathway is about observable implications for learning as a dependent variable. In the analytical framework we presented, the value of two variables (problem tractability and certification of actors) will determine the prevailing learning type and the associated style of interaction between policy actors (e.g., partisan mutual adjustment or communicative rationality, rule and procedure‐driven or evidence‐based driven), the nature of policy fora (idea‐labs, arenas, participatory venues, or consultation and advice bodies established by law and parliamentary procedure), and so on. As outlined, it also predicts each of the 16 roles on the basis of two additional variables—control of learning content and learning objectives (as in Figure 3). Further, we have implications from micro‐foundational studies. Under conditions of extreme surprise, we should observe contingent learning first, followed by inferential learning. This is a proposition that can be tested in cases of acute crisis, and more precisely in intra‐crisis periods (Kamkhaji & Radaelli, 2016), and crisis management capacity‐building (e.g., Schwarzer, 2015).

Additionally, authors like Boswell (2008) have put forward propositions about how organizations use the knowledge they have learned, depending on the features of the policy area (namely, the acknowledgement, in a given policy domain, of epistemic uncertainty versus the acceptance of technical, science, and evidence‐based modes of settlement) and the type of organization. Most interestingly perhaps, Boswell argues that an organization that does not control its output will use knowledge to legitimize policy. Organizations like the European Commission, which depends on the bureaucracy of the member states of the European Union for implementation and ultimately output, will exhibit more legitimizing than instrumental usages. These types of propositions can be tested across a large number of policy sectors and types of organizations. They also invite more integration between policy learning and two research fields we do not cover in this paper, but have many points of contact: ideational politics and knowledge utilization (Freeman & Sturdy, 2014; Radaelli, 1995).

Turning to the third pathway, we look at observable implications of the type “if this learning occurs … then we should observe this effect on the dependent variable.” The classic dependent variable for learning, but also for other frameworks, is policy change. Actually the relationship between learning and change is qualified. An individual can learn without necessarily being able to bring about change within an organization. A single organization can learn but change may not materialize because of institutional inertia, complexity, veto players, and other factors. A whole system may learn without having the necessary capacity to change.

Add that change has different properties depending on the variety of learning we take into account. Under “bargaining” change will take the form of an agreement or compromise among the actors interested. Whether this necessarily leads to major policy change is an open question. It depends on resources, capacity, de‐coupling between the compromise and the actions, and so on. The old argument that many incremental changes make a big change applies. In “epistemic” types change will involve an alteration of priors on the basis of evidence. Yet again, this may or may not trigger change. Certainly epistemic networks have generated changes in domains like climate change, international tax evasion, and poverty—yet the degrees of implementation we have seen in these three international policy domains vary markedly.

In “reflexivity,” we find the mechanism for major policy change. Actually authors working on experimental learning (Sabel & Zeitlin, 2008) and social learning (Hall, 1993) point to the inclusive properties of this mode to draw conclusions about societal‐level change—or at least major paradigmatic changes in a policy field (like macro‐economic and monetary policy for Hall, or sectors like energy and employment for Sabel & Zeitlin). Some of these propositions about the effects of reflexivity have now been tested across sectors with corroborating evidence (Sabel & Zeitlin, 2012; Zeitlin, 2015).

For “hierarchy” we have a full set of propositions concerning coordinated action in certain types of multi‐level governance settings, like the ones dominating the European Union and German federalism. Fritz Scharpf has generated a causal model of how joint‐decision systems hinder policy change. Take a multi‐level governance system where some decisions are taken at the higher federal level. When these decisions are taken by the participating actors (goverments in the EU, Länder in German federal decisions) without a principle of representation that filters out the immediate interests of the lower units and there is a formal or informal unanimity rule, then decisions are suboptimal. Policies that are already in place are hard to reverse, and new decisions cannot be taken at the federal level—whilst the lower units have lost competence for these decisions. This is the joint‐decision trap. Recent work, however, has shown how the joint‐decision trap can be relaxed (Falkner, 2011). In a revealing footnote in one of his joint‐decision trap articles, Scharpf remarks about the difference between his Coasian approach and spatial voting theory. He observes that the Coasian theory he adopts includes policy learning: “spatial voting theory, in contrast, ignores the possibility of policy learning and takes fixed preferences over strategies” (Scharpf, 2006, p. 850, footnote 4, note that here Scharpf uses “strategies” as proxy for “policies”).

The joint‐decision trap would take us into a long discussion. All we can say here is that joint‐decision traps, and escape routes from them, are intimately connected to learning processes, although the explicit derivation of observable implications of learning for change is rare in extant literature.^1^ Essentially, some forms of hierarchical learning can be compatible with rational choice institutionalism and can explain how actors find escape routes from joint‐decision traps. Other types of learning are obviously empirically possible, and grant additional ways to escape traps. However they are not compatible with the assumptions of rational choice institutionalism and should not be considered “extensions” of Scharpf's model. Simply, they belong to other types of explanations. This leads us to an important corollary: there is an affinity between rational choice institutionalism and two varieties of learning—bargaining and hierarchy—whilst epistemic and reflexive learning lend themselves quite naturally to sociological institutionalism and constructivism.

To sum up, learning has developed observable implications that add to existing frameworks on the policy process. It has also provided testable propositions about how to identify one type of learning or another, or sequences of learning. Here, however, the literature has just begun to operationalize concepts, hence measurement is a challenge. When we turn to learning as an independent variable, we have conjectures about when we should expect or not expect policy change, and its causal drivers under different modes of learning. Yet again, the literature is somewhat under‐developed here.

## Normative Dimensions—Connecting Learning to Democracy

As well as offering a positive theory explaining large elements of the policy process, claims for “promising” framework status are greatly enhanced by those approaches that contain normative elements (Sabatier, 1999, p. 8; 2007, p. 8). The assumption that learning is a “good thing” is implicit in much of the literature. But as mentioned, learning is not always desirable. We can think of individuals and organizations learning something that is dysfunctional and/or normatively unacceptable in terms of democratic accountability or legitimacy. That said, the framework is not normative in the tradition of social constructivism of Schneider and Ingram (1997), for example. Yet, a non‐normative framework can still have prescriptive implications. Indeed, deLeon and Weible (2010) outline no fewer than six pathways to link policy process research with the improved democratic practices so passionately argued for by Lasswell (1951). The first three are what they term as the “oversubscribed” strategies used by policy theorists to make their work relevant beyond academia. We relate them to the varieties of learning framework in turn.

1. *Reliance on the policy implications* that can be drawn from policy research (deLeon & Weible, 2010, pp. 25–26) is the prescriptive dimension most connected to varieties of learning, thus far. Using the concepts of functional and dysfunctional policy learning, the framework has been extended to identify the conditions for efficient learning in each of the four modes. Here the prescriptive reasoning is confined to the logic of the framework itself. Table 1 outlines the scope conditions for dysfunctional and efficient learning. One of the advantages of these underpinnings is that it lends greater precision to our reasoning. Recall deLeon and Weible (2010) chide researchers for being too ambiguous in the policy implications they often draw (pp. 25–26). This has been empirically explored in two empirical cases—the policy failure of bovine tuberculosis in England (Dunlop, 2017b) and the crisis of sovereign debt in the Eurozone (Dunlop & Radaelli, 2016). Both cases demonstrate that actors and institutions learn how not to comply. They also learn how to get trapped in their habits (heuristics) and competences, or learn “bad lessons.” Taking the Euro example, Dunlop and Radaelli (2016) draw on varieties of learning to explore design, and precisely how the institutions and policy instruments of the European Union should be designed to generate socially inclusive, legitimate, and accountable learning. Or, put differently, if an organization is not learning in the right mode, we can identify what design features would make it learn in a more desirable mode.
2. *Reliance on normative theories* embedded in policy theories (deLeon & Weible, 2010, pp. 26–27) is the second oversubscribed strategy for connecting with democracy. That is not the case for this approach. Indeed, it is one of the varieties of learning framework's biggest areas of developmental need. That learning can be concentrated in one mode or is not necessarily efficient or normatively desirable raises issues of legitimacy and accountability. Learning modes generate power shifts. Take for example the scenario where learning is limited to the epistemic realm. This may lead to more than the dysfunctional groupthink to technocratic domination—bolting on ideas from normative political theory may help us determine whether we can consider this rule without justification or legitimate and accountable.
3. *Reliance on political advocacy* directed by our frameworks (deLeon & Weible, 2010, pp. 27–28). Outlining the conditions for functionality and dysfunctionality of learning offers inspiration to actors seeking an advocacy role by making their policy engagement more fruitful. A recent application of this has been made to the world of experts. Using the varieties of the learning framework to outline the probable worlds of policy learning scientific advisers can inhabit, Dunlop (2014) then extends the framework using knowledge utilization literature to postulate the types of “possible” expert personae that are required to function and flourish in each learning mode. Policy experts or “issue advocates” (Jenkins‐Smith, 1990) can achieve more effective engagement and impact by adopting a mode of engagement to match the learning setting they find themselves in (Dunlop, 2014).

In sum, while we appear some way off from a policy learning sciences of democracy, we have identified some promising beginnings. One final element to consider: varieties of learning includes four types and 16 subtypes. In some of the 16 cells, the reflection on the normative implications has already been deep. Think for example of the field of experimental governance (Sabel & Zeitlin, 2008), where the key authors are engaged in a conversation with constitutionalism and democratic theory (Cohen & Sabel, 1997)—to the point that experimentalism is seen as an embodiment of a new form of democratic governance.

## Conclusions

We have appraised the field of policy learning as a promising analytical framework of the policy process. It is useful to repeat that we have chosen to focus on the varieties of the learning approach not because it is better than others, but because it allows us to treat in a single analytical template a vast portion of the field, as shown by its assemblage of four types and 16 subtypes.

So, does policy learning meet the standards of an analytical framework? We have considered assumptions and micro‐foundations, the conceptual apparatus, observable implications, and the normative usages. Our analysis shows that policy learning meets these standards, although with variation. We point to specific research questions where research should be intensified. More work has been done on assumptions and concepts, less on normative appraisal. On observable implications we have cumulated more findings and causal relationships on learning as a dependent variable than on learning as an independent variable.

In fact, there are several conjectures about why and how learning as an independent variable can affect or hinder change, but we need to improve on operationalizations and measures that allow researchers to go confidently in the field. We have a set of conjectures waiting for confutation or corroboration. In short, there is a lot of work in progress. The same can be said of normative analysis.

One point we wish to make is that policy learning is not stuck to where we were in the 1990s. It is a progressive research agenda, with solid ontological assumptions, where new conjectures and causal relations have been theorized and are beginning to be tested. This progressive character of the agenda underpins the enthusiasm evidenced by so many special issues on learning produced in recent years. In a sense, the field we have considered is both classic (as mentioned we can trace its origin back to very classic, foundational articles) and able to attract new empirical and theoretical work. All this is a signal of vitality.

Certainly, the field has many limitations—the major is that after so many years from the intuitions of Peter Hall (1993), Bennett and Howlett (1992), May (1992), we are still struggling with the causal relation between learning and change. Possibly what we are finding out in terms of micro‐foundations and, turning to another dimension, the Coleman's “bath‐tub” structure of causality (Dunlop & Radaelli, 2017), will illuminate the way ahead. As mentioned, presumably more leverage will need a closer integration among policy learning, ideational analysis, and knowledge utilization (on the latter, see the attempts made by Schrefler, 2010, and Radaelli, 2009). We also have to be clearer on the causal relations leading to nonlearning, endarkenment, and the pathology of learning—another area where there is a lot of exciting, fresh work but not much cumulative empirical knowledge so far.

With all these limitations, we have the impression that policy learning fares reasonably well as an analytical framework of the policy process. The next step is to measure “how well” with a systematic comparison with other, more established, frameworks.

## Note

1 In Falkner's edited collection on decision traps see chapter 10 on learning as pathway out of stalemate (Alecu de Flers, Chappell, & Müller, 2011), and in the concluding chapter Scharpf attends to (instrumental) policy learning in another of his illuminating footnotes (Falkner, 2011; Scharpf, 2011, p. 233, footnote 18).
